# Can a central pattern generator produce multiple motor patterns? Modeling scratch rhythms in turtle

**DOI:** 10.1186/1471-2202-13-S1-P52

**Published:** 2012-07-16

**Authors:** Abigail Snyder, Jonathan Rubin

**Affiliations:** 1 Department of Mathematics, University of Pittsburgh, Pittsburgh, PA 15213, USA

## 

**Figure 1:**
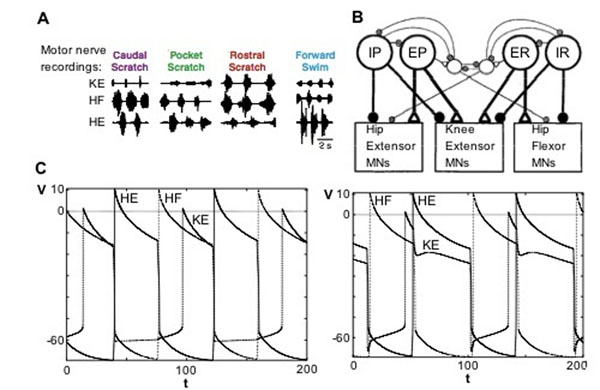
**A. E**xample recordings of fictive scratching and fictive swimming [2]; **B.** The proposed CPG [1]; **C.** Results of simulation for Rostral (left) and Pocket (right) scratch.

A central pattern generator (CPG) is a population of neurons producing rhythmic or repetitive behavior (i.e. scratching, walking, masticating) without requiring rhythmic input to the population. Turtles are observed to produce several rhythmic motor patterns in response to stimuli, in particular rostral scratch, pocket scratch, caudal scratch, and forward swim (see Figure 1A) [1], [2]. The rostral scratch and pocket scratch rhythms are created through the activity of three motoneurons: Hip Extensor (HE), Knee Extensor (KE) and Hip Flexor (HF). A CPG (see Figure 1B) for rostral scratch and caudal scratch has been proposed, featuring overlapping populations of spinal neurons such that each spinal neuron projects to each motoneuron to produce both scratch rhythms via changing inputs [1]. We implement the CPG as a system of relaxation oscillators. The system successfully reproduces the desired rhythms (see Figure 1C). We also consider a dynamical systems approach to determine the mechanisms underlying rhythm generation, seeking the minimal network necessary to produce both rhythms. Additionally, we numerically explore the role of model parameters and present sufficient conditions on model parameters to produce both rhythms.
